# The crossroads between cancer stem cells and aging

**DOI:** 10.1186/1471-2407-15-S1-S1

**Published:** 2015-01-15

**Authors:** Sara Santos Franco, Hadas Raveh-Amit, Julianna Kobolák, Mohammed H Alqahtani, Ali Mobasheri, András Dinnyes

**Affiliations:** 1 Szent István University, Gödöllö 2100, Hungary; 2 Biotalentum Ltd., Gödöllö 2100, Hungary; 3 Center of Excellence in Genomic Medicine Research (CEGMR), King AbdulAziz University, Jeddah, 21589, Kingdom of Saudi Arabia; 4 Department of Veterinary Preclinical Sciences, School of Veterinary Medicine, Faculty of Health and Medical Sciences, University of Surrey, Duke of Kent Building, Guildford, Surrey, GU2 7XH, United Kingdom; 5 Department of Farm Animal Health, Faculty of Veterinary Medicine, Utrecht University, Utrecht 3584 CL, The Netherlands

**Keywords:** Stem cells; aging; cancer stem cells; malignant transformation; tumorigenesis.

## Abstract

The cancer stem cell (CSC) hypothesis suggests that only a subpopulation of cells within a tumour is responsible for the initiation and progression of neoplasia. The original and best evidence for the existence of CSCs came from advances in the field of haematological malignancies. Thus far, putative CSCs have been isolated from various solid and non-solid tumours and shown to possess self-renewal, differentiation, and cancer regeneration properties. Although research in the field is progressing extremely fast, proof of concept for the CSC hypothesis is still lacking and key questions remain unanswered, e.g. the cell of origin for these cells. Nevertheless, it is undisputed that neoplastic transformation is associated with genetic and epigenetic alterations of normal cells, and a better understanding of these complex processes is of utmost importance for developing new anti-cancer therapies. In the present review, we discuss the CSC hypothesis with special emphasis on age-associated alterations that govern carcinogenesis, at least in some types of tumours. We present evidence from the scientific literature for age-related genetic and epigenetic alterations leading to cancer and discuss the main challenges in the field.

## Introduction

The cancer stem cell (CSCs) paradigm has started a new era in cancer research with significant implications for the formulation of future clinical therapeutics [recently reviewed in [1,2]]. This hierarchical model for cancer, unlike the classical stochastic model, supports the existence of CSCs, or tumour-initiating cells, that are responsible for tumour formation, maintenance, growth and metastasis [3,4]. Although CSCs were identified in solid and non-solid tumours, our current knowledge concerning the origin of these cells and the processes leading to their formation is limited due to the complexity of the experimental approaches that will be required to provide data of sufficient substance to support this hypothesis [5,6]. Given the stem cell-like characteristics of CSCs, it has been proposed that CSCs originate from adult stem cells, progenitor cells or differentiated cells that have acquired ‘stemness’ properties [3,6-8]. Although all three cell sources have the capacity to be genuine CSCs, in our view, adult stem cells appear to be the most probable target for malignant transformation, generating cells with stem-like properties and tumorigenic potential [6,9,10] and accounting for cancer as a ‘stem cell disease’ [9]. Unlike adult stem cells, progenitor cells and somatic cells are more lineage-committed and comprise reduced proliferation potential, thus requiring additional alterations to re-acquire the self-renewal potential [3,6,9].

One of the major drivers of malignant transformation is aging [11-15]. With age, all cells, including stem cells, accumulate genetic and/or epigenetic alterations, which affect the cellular, molecular and physiological functionality of tissues [13-16]. The consecutive deterioration of tissues is an important risk factor for the origin of age-related chronic diseases in humans, such as diabetes, cardiovascular and neurodegenerative diseases, etc. Age-associated effects may also lead to a higher risk of tumorigenesis, when in concomitant with deregulated cell signalling and changes in the microenvironment of stem cells. These effects increase the resistance to cellular senescence and programmed cell death and may ultimately lead to the transformation of stem cells into CSCs. Although direct evidence for this concept is missing, it is consistent with the notion that CSCs originate from adult stem or progenitor cells. Further research is needed to better understand the age-associated alterations involved in the generation of CSCs and advances in this area are expected to shed new light on cancer diagnosis and treatment. The present review focuses on the relationship between aging and cancer stem cells and aims to explore the latest advances in the field.

## The CSCs model

In the last few decades, the concept that tumours may comprise a rare population of cells with self-renewal, proliferation and differentiation capacities, resembling stem cells, has emerged. The first evidence for CSCs came from the identification of leukemic stem cells (LSCs), which are the cancer-initiating cells of the hematopoietic system [17]. LSCs were identified as a population of cells expressing the CD34^+^CD38^-^ cell surface markers with a uniquely oncogenic phenotype characterized by their ability to initiate leukaemia in non-obese diabetic mice with severe combined immunodeficiency disease (NOD/SCID mice) [18]. The development of fluorescence-activated cell sorting (FACS), together with the establishment of specific cell surface markers, permitted the subsequent identification of CSCs in solid tumours [7]: breast (CD44^+^CD24^-/low^) [5], prostate and ovarian (CD44^+^) [19,20], brain and lung (CD133^+^) cancers [21,22], and others [23-25]. In addition to malignant tumours, tumour stem cells have been identified in benign tumours, as demonstrated recently by Xu and colleagues (2009). These cells were isolated from pituitary adenoma and exhibited similar characteristics to multipotent stem-like cells, such as self-renewal capacity, multi-lineage differentiation, sphere formation, resistance to chemotherapy, and tumour formation in NOD/SCID mice [26]. The presence of CSCs in both benign and malignant tumours demonstrates the ability of these cells to facilitate tumour formation and support carcinogenesis.

**Figure 1:**
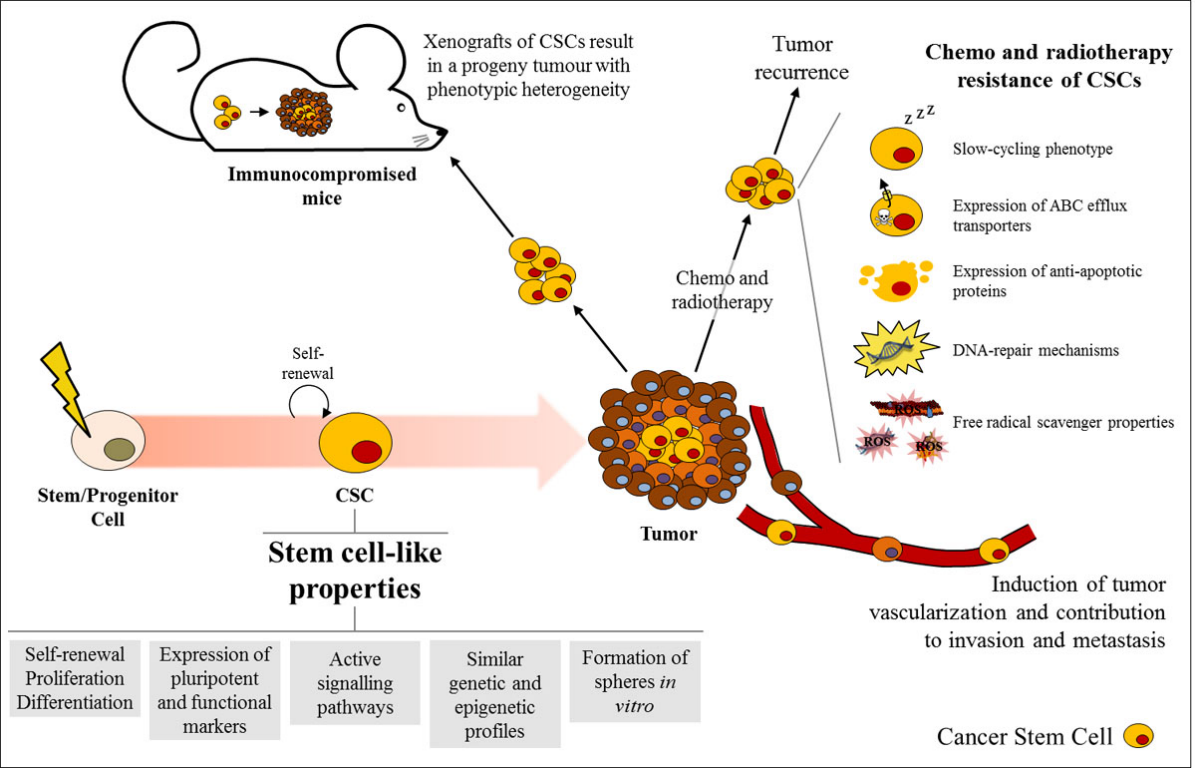
**Characteristics of CSCs.** CSCs are tumour-initiating cells that may result from malignant transformation of stem/progenitor cells, instigating the tumorigenic process. CSCs have been described to possess stem-like properties, such as self-renewal, proliferation and differentiation abilities, expression of pluripotent (e.g. Sox2, Oct4, Nanog) and functional (e.g. ALDH1, CD133^+^, CD34^+^CD38^-^) markers, active signalling pathways (e.g. Notch, Hedgehog, Wnt), genetic and epigenetic profiles similar to stem cells, and capacity to form spheres *in vitro*. CSCs can be efficiently detected when injected in immunocompromised mice, as these cells, through their self-renewal and differentiation potential, give rise to a tumour with phenotypic heterogeneity. Tumorigenesis is followed by angiogenesis and by the invasion and metastatic stages, as part of the disease progression. Indeed, CSCs have been associated with the induction of tumour vascularisation through the expression of vascular-related factors and by their contribution to metastasis through the induction of the EMT program. Their resistance to chemo and radiotherapies is clinically important as most anticancer agents target the tumour bulk but not the CSC population. The resistance ability of these cells may be associated with their slow-cycling phenotype, and/or expression of efflux transporters, anti-apoptotic proteins, DNA-repair mechanisms, or of free radicals scavengers.

The identification of CSCs led to the characterization of their stem cell-like properties (Figure 1), consistent with their ability to cause tumour formation and recurrence [27,28]. By definition, normal stem cells give rise to the cellular components of an organ by asymmetric division mediating their self-renewal and differentiation potential [6]. Similarly, CSCs mimic this process by promoting aberrant organogenesis in a hierarchical-mode, in which a phenotypically heterogeneous progeny at different levels of differentiation and proliferation is formed [10]. The stem-like characteristics of CSCs include the expression of pluripotent markers such as Sox2, Oct4, and/or Nanog [27,28] and of functional markers, like ALDH1, CD133^+^ (as lung stem cells), or CD34^+^CD38^-^ [as hematopoietic stem cells (HSCs)] [9,29]. Furthermore, CSCs resemble normal stem cells by sharing active signalling pathways, such as Notch, Hedgehog, and/or Wnt [3,30], similar genetic and epigenetic profiles [31], and aptitude to form spheres *in vitro*[27,32].

**Figure 2:**
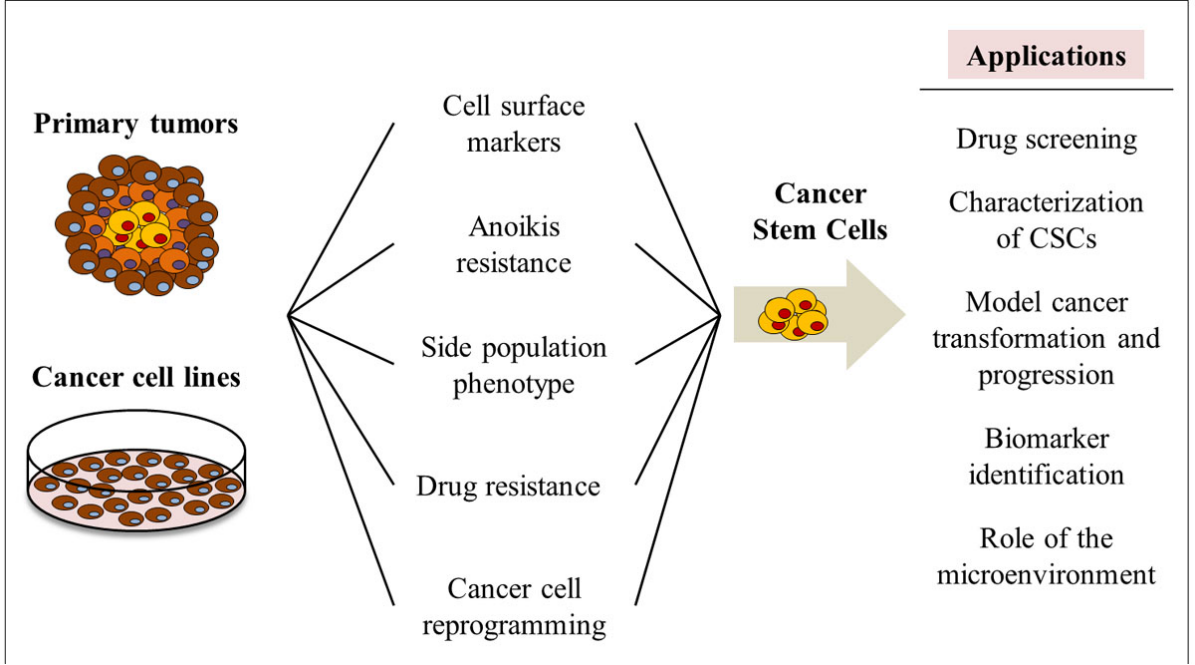
***In vitro* models of CSCs and their applications.** Different *in vitro* models of CSCs have been created in an attempt to allow a better understanding of the properties of these cells but also of the cancer biology. In addition, these models have been employed in drug screening assays but also in the identification of biomarkers associated with different stages of neoplasia and its recurrence after therapy. Generally, CSCs can be isolated from primary tumours and cancer cell lines based on definite properties, such as expression of specific cell surface markers (e.g. CD44^+^, CD133^+^, CD34^+^CD38^-^), resistance to anoikis or to drugs, or possess of a side population phenotype. Furthermore, recent reports have demonstrated the generation of CSC-like cells through the reprogramming of cancer cells from both primary tumours and cancer cell lines.

The isolation and maintenance of CSCs advanced our understanding of cancer initiation and progression, resulting in *in vitro* models to characterize these cells, model cancer transformation and progression, study the effect of the microenvironment [33], screen for CSC-specific drugs [34,35], and identify biomarkers for the onset, progression of cancer and its recurrence after therapy [36] (Figure 2). CSCs can be isolated from cancer cell lines or primary tumours based on the i) expression of surface markers [37,38], ii) detection of the side population [39], iii) anoikis resistance [40], or iv) drug resistance [41]. However, the low frequency of CSCs in primary tumours and the difficulty to stably maintain these cells *in vitro* makes some of these systems difficult to use. To overcome these issues, *in vitro* models of cancer stem-like cells have been developed recently. Chen and colleagues (2012) developed a CSC model from mouse induced pluripotent stem cells (miPSC) cultured in a medium simulating the tumour microenvironment [35]. Sachlos *et al* (2012) established a valuable screening assay for CSCs-targeting drugs using neoplastic human pluripotent stem cells (hPSCs) [34]. Additionally, several reports demonstrated that cancer stem-like cells can be obtained by the reprogramming of cancer cells [42,43] and primary tumours [36] to iPSC-like induced pluripotent cancer cells (iPCs). Unfortunately, this process is time-consuming and its efficiency is even lower than the reprogramming of non-tumorigenic somatic cells. The stem-like characteristics of iPCs were validated through the expression of pluripotent markers, such as Oct3/4, Sox2, or Nanog, as well as SSEA-4, Tra-1-60, or Tra-1-81; and the capacity of iPCs to form the three germ layers via embryoid bodies *in vitro* and teratomas *in vivo*[4,27,28,42-44]. Furthermore, iPCs are resistant to several anticancer drugs [43], express CSCs markers, like CD133 [28], and demonstrate tumorigenic and metastatic properties *in vivo*[28,36].

Based on the tumorigenic potential and self-renewal properties of CSCs, these cells can be easily detected by serial transplantation in immunocompromised mice, while the progeny tumour represents the phenotypic heterogeneity of the parental tumour [10] (Figure 1). Conversely, non-tumorigenic cells have lower proliferative and anti-apoptotic capacities, as confirmed by their decreased Hoechst dye efflux or aldehyde dehydrogenase activities and do not form tumours *in vivo*[39]. This notion is consistent with the positive correlation between the higher incidence of differentiated cancer cells in a given tumour and favourable prognosis [3,6,7]. Differentiation of CSCs, or so called “differentiation therapy”, was therefore proposed as a novel approach to eliminate CSCs [45,46] using different compounds, such as all-trans retinoic acid (ATRA), an inducer of the promyelocytic differentiation used for the treatment of acute promyelocytic leukaemia [47], and suberoylanilide hydroxamic acid (SAHA), a histone deacetylase inhibitor and differentiation inducer of human breast and cancer cells [48].

CSCs are involved in cancer progression and metastasis [49] (Figure 1). These cells induce tumour vascularisation by promoting angiogenesis, through the expression of vascular-related factors, e.g. VEGF and PD-ECGF [50], and by stimulating the incorporation of CSC-derived endothelial cells into newly formed capillaries [51]. Tumour vasculature is not only important for supplying blood to the tumour but also for the proliferation and maintenance of CSCs [51], resulting in a “vicious cycle” contributing to cancer progression. Furthermore, CSCs may contribute to invasion and metastasis by acquiring migratory properties through the induction of the epithelial to mesenchymal transition (EMT) program [14,49]. This concept was supported by the activation of β-catenin and the low levels of E-cadherin in stem-like tumour cells at the tumour-host interface [52] and the induction of mesenchymal markers in CSCs, such as vimentin and N-cadherin [28]. Moreover, tumours can become metastatic as a result of the accumulation of extra mutations and/or epigenetic modifications within the CSCs [2,46].

The formation of CSCs in tumours and their maintenance, angiogenic support and subsequent metastatic potential are clinically important especially since most current antitumor therapies target the bulk tumour and not CSCs. Moreover, the resistance of CSCs to chemotherapy and radiotherapy supports their association in tumour recurrence after therapy [35,53-55] (Figure 1). This is consistent with the increased expression of ABC efflux transporters [56,57], like P-glycoprotein and/or ABCG2, and/or anti-apoptotic proteins (e.g. Bcl-2, survivin, and NF-κB) [3,14], and with the higher DNA-repair capacity [14], elevated free radical scavenger properties [58], and/or slower proliferation potential related to a slow-cycling state (G_0_ phase) of these cells [53]. Overall, it is critical to develop novel therapies that are adjusted to effectively eliminate CSCs and tumour recurrence. However, considering that normal stem cells and CSCs are very similar, effective chemotherapeutic agents must selectively target CSCs but not normal stem cells [34,53].

## Origin of CSCs: Adult stem cells *vs* progenitor cells

Do CSCs originate from adult stem or progenitor cells? Given that these cells represent a rare population within a tissue, similarly to CSCs in the tumour, makes them difficult to study [10]. Furthermore, the process in which an adult/progenitor cell undergoes malignant transformation into a CSC is very complex and may involve multiple stages. Nevertheless, strong evidence suggests that most tumours originate from CSCs through neoplastic alterations of adult stem or progenitor cells [2,9,59].

Adult stem cells constitute small populations within the tissues that are important for tissue homeostasis and regeneration by replacing senescent cells and those lost as a consequence of tissue injury [11]. Through asymmetric division, stem cells support their self-renewal while maintaining their tissue-specific differentiation capacity [13]. Although HSCs were the first adult stem cells to be described, the existence of adult stem cells have been confirmed in other tissues, such as heart [60], lung [61], brain [62], skeletal muscle [63], kidney [64], and others [65-67].

Adult stem cells have a longer lifespan than progenitor and somatic cells; long enough to allow the accumulation of age-associated genetic and/or epigenetic alterations responsible for malignant transformation into CSCs [2,3,10,14,15,68,69]. For this reason, during chronological aging, adult stem cells are more likely to be the target of alterations that may lead to the formation of CSCs. This notion is further supported by the observation that progenitor cells lose their self-renewal property during commitment, an important capacity that should be re-acquired in order to undergo transformation [70]. Adult stem cells can self-renew and thus require fewer mutations and/or epigenetic modifications to undergo neoplastic transformation than progenitor cells [3]. Progenitor cells may gain stem cell-like characteristics through the activation of self-renewal-related genes through *de novo* mutations [70] or gain of mutations of adult stem cell’s origin [3], or via EMT induction [2,71].

Since hematopoietic lineage markers are well known, leukaemia has become an important model for the study of the cell origin of LSCs. Nevertheless, LSCs’ origin is still controversial [3,6]. Many reports support that leukaemia arises from malignant HSCs [3]. A study on patients with acute myelogenous leukaemia (AML) detected different cell types of the myeloid and lymphoid lineages expressing the *AML1/ETO* transcript. The authors suggested that the acquisition of the genetic translocations had occurred at the stem cell stage, as these cells were able to differentiate into B cells and cells from the myeloid lineage, and some of which acquired additional mutations that ultimately resulted in AML [72]. These results are further supported by the observation that LSCs are CD34^+^CD38^-^, similarly to normal primitive cells, supporting that HSCs are a possible target for transformation into LSCs [73]. Chronic lymphocytic leukaemia (CLL), a malignancy involving mature B lymphocytes, was demonstrated to be caused by the accumulation of oncogenic episodes within the HSC population [74].

The acquisition of self-renewal capability in progenitor cells may induce leukaemia, as observed by the expression of the MLL-ENL [75], MOZ-TIF2 [76], and MLL-AF9 oncogenes, or by the activation of β-catenin [77] in progenitor cells of the hematopoietic system. However, when the same oncogenes were expressed in HSCs, fewer transformed cells were required for leukaemia induction *in vivo* and the tumours induced by these cells were more heterogeneous [2]. These results indicate that progenitor cells require self-renewal properties in order to be transformed into LSCs, a mechanism that already exists in HSCs. In addition to leukaemia, CSC’s origin has been determined in solid tumours such as gliomas. When *Nf1* and *p53* gene mutations were introduced into neural stem cells (NSCs), no abnormalities were observed, except when these alterations were present in the oligodendrocyte precursor cells (OPCs), as confirmed by the occurrence of glioma [78]. Instead, when telomerase expression was induced in adult mesenchymal stem cells, neoplastic transformation occurred and tumour initiation was observed [79]. Furthermore, evidence has shown that the induction of oncogene expression or abrogation of tumour suppression genes induces the transformation of stem cells into CSCs, leading to tumour development [80-84]. Morrison and colleagues (2008) studied the tumours of the peripheral nervous system (PNS) and demonstrated that the absence of Nf1-deficient neural crest stem cells in postnatal mice, supporting that the PNS tumours did not originate from these stem cells but rather from differentiated glia [8]. Another study showed that the lack of *INK4A* and *ARF* genes and the activation of the EGFR pathway in both NSCs and differentiated astrocytes induced gliomagenesis [85].

Further studies are necessary to improve our knowledge regarding the origin of CSCs, as these cells seem to result from the transformation of adult stem cells, progenitor and/or differentiated cells. Due to the longer lifespan of adult stem cells, these cells are more likely to be the target for the accumulation of genetic and/or epigenetic events that may induce the first steps of tumorigenesis and carcinogenesis. Furthermore, the self-renewal and differentiation properties of stem cells allow the accumulation of mutations and other alterations to the downstream progeny during the lifespan of an organism [68], explaining why some types of progenitor and differentiated cells can give rise to CSCs.

## Aging and transformation of stem/progenitor cells into CSCs?

**Figure 3:**
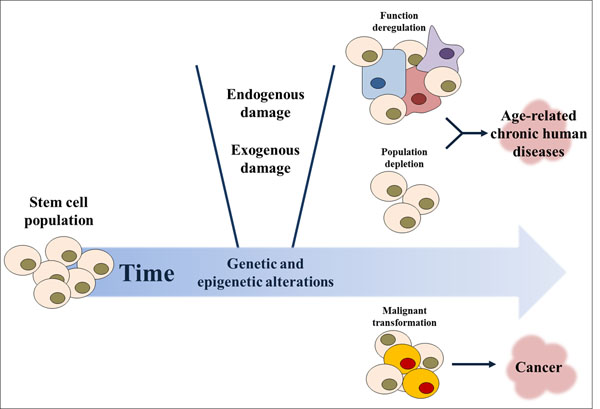
**Age-related effects in the stem cell population.** Stem cell populations, and their progeny, are subjected to age-effects that are related to the decline in tissue regeneration and homeostasis and consecutive onset of chronic human diseases, such as neurodegenerative disorders, heart failure, and diabetes. Deregulation of stem cell function or decrease of their population, due to cell senescence and apoptotic death, are the main factors for these age-related observations. Furthermore, malignant transformation of stem/progenitor cells may occur, leading to tumour formation and carcinogenesis. Indeed, the aging process is associated with the accumulation of genetic/epigenetic alterations within the stem/progenitor cell populations, together with telomeric DNA reduction, DNA repair deficiency, and chromosome rearrangements that may induce this process. During time, endogenous (e.g. ROS, RNS, spontaneous hydrolysis, alkylation, replication errors, telomere shortening, etc.) and exogenous (e.g. UV light, chemical, chemotherapeutic and radioactive compounds, X-rays, ionizing radiation, etc.) damage may occur, thus affecting the stem cell population.

Mammalian aging is associated with a reduction in organismal functionality and this is accompanied by an age-related decline in tissue regeneration and homoeostasis [11,13,14,68]. Due to its complexity, the biological mechanisms underlying the aging process are still not well known [68]. Diverse theories have been proposed in an attempt to explain this issue [11], including the mutation accumulation theory, suggested in 1952 by Medawar [86], and the antagonistic pleiotropy theory, suggested by Williams in 1957 [87]. Later on in 1977, Kirkwood published the disposable soma theory, suggesting that an organism has a limited amount of energy that should be divided between the non-reproductive part of the organism, or soma, and the reproductive part, or germ line. Lifespan is controlled by an energetic balance needed to allow the organism to repair age-related damage with minimal impact on reproductive capacity [88]. Immortal germ cells must resist stress to be able to transmit genetic information to subsequent generations with high accuracy and reliability [11]. In this context, adult stem cells can be considered part of the somatic content of an organism as they are not immortal and have a finite replicative lifespan, in contrast to embryonic stem cells (ESCs) and germ cells [89]. Thus, this theory predicts that adult stem cells may be subjected to age-related alterations, such as telomeric DNA reduction, DNA repair deficiency, chromosome rearrangements, genotoxic effects, and accumulation of genetic/epigenetic alterations [14] (Figure 3). Indeed, it is widely accepted that aging is associated with stem cell pool depletion, as a consequence of cellular senescence or apoptotic death, and/or their reduced functionality, leading to reduced tissue regeneration potential and impaired homeostasis [11,89,90]. These effects are associated with the onset of age-related human diseases, such as neurodegenerative disorders, heart failure, arthritis, diabetes, etc. [13,68,91] (Figure 3). Furthermore, the transformation of stem/progenitor cells into CSCs can be induced with time, leading to tumour formation and carcinogenesis [2,3,10,14,15,68]. In fact, cell senescence and apoptosis are important mechanisms for the organism as a defence from tumour formation [7,11,13,14,92,93]. This review has focused on aging-associated tumorigenesis.

Some types of cancer can be viewed as age-related disorders associated with genetic and/or epigenetic alterations of stem/progenitor cells along with the deregulation of their microenvironment [14,68,94-99]. In the case of HSCs, a direct link was established between the age-related loss of lineage specificity and regenerative capacity [95], and the onset of leukaemia [94-97]. This relationship is supported by the expression of leukaemia-related genetic [95] and epigenetic alterations [100] in aged HSCs, associated with an aged bone marrow microenvironment that allows the expansion of LSCs [97]. In addition to leukaemia, adult stem/progenitor cells deficiency and loss of their function during aging have been associated with the onset of non-hematopoietic tumours, such as skin and brain cancers. For example, the resistance of stem cells to cell death and senescence was shown to be mediated by age-associated self-renewal deregulation and genetic and/or epigenetic alterations, inducing their transformation into CSCs and skin tumour [98]. Moreover, aged NSCs have been associated with tumour formation and neurodegenerative disorders, as a consequence of their impairment and inability to perform tissue homeostasis [99].

## Age-associated genetic events and generation of CSCs

During the lifespan of an organism, stem cells are subjected to DNA damage, such as base modifications, inter or intrastrand crosslinks, DNA strand breaks, etc. Such damage can result from the effect of endogenous [e.g. reactive oxygen species (ROS), reactive nitrogen species (RNS), spontaneous hydrolysis, alkylation, replication errors, etc.] and exogenous (e.g. UV light, chemical, chemotherapeutic and radioactive compounds, X-rays, ionizing radiation, etc.) genotoxic agents, telomere shortening, and spontaneous degradation of DNA, leading to genome instability [68,101,102] (Figure 3). Unlike damaged proteins, lipids and RNA, which can be replaced, damaged DNA cannot be substituted and it is easily heritable to daughter cells. To ensure that the genomic integrity of stem cells is well preserved throughout the lifespan of an organism, these cells must resist damage from noxious external stimuli and injuries. DNA damage response pathways are highly important for stem cells, as shown by the exceptional capability of stem cells to repair DNA, in contrast to other cell types [101], and by studies with DNA repair-deficient mice that showed a senescence- and apoptosis-related depletion of the stem cell population [103]. Nonetheless, age-dependent reduction in DNA damage repair pathways occurs in stem cells and may contribute to their dysfunction as well as malignant transformation and tumorigenesis [104,105].

Stem cells may possess an additional cytoprotective system to protect against the acquisition of genetic and epigenetic modifications in their genomes. The immortal strand hypothesis states that, during the asymmetric division of a stem cell, the segregation of its DNA takes place in a non-random manner as it depends on the DNA strand’s age. In fact, the new stem cell acquires the oldest template from the DNA replication and the other daughter cell that undergoes differentiation obtains the newly synthesized strand. The stem cell pool includes the DNA strands with fewer mutations acquired during DNA replication and the most similar to the original cell population generated during development [106]. Although evidence exists to support this theory in many types of stem cells, the biochemical mechanisms responsible for this event are still under investigation [106-108].

Although stem cells possess efficient systems for protection from DNA damage, these systems are not perfect and may be overwhelmed. Therefore genetic/epigenetic alterations can elude and accumulate with time within cells [68]. This observation was confirmed by a study from Welch *et al* (2012), revealing that HSCs and progenitor cells from healthy individuals accumulate mutations with age, 0.13 exonic mutations per year of life [15]. Though most of these background mutations are not relevant for the initiation of leukaemia, few relevant mutations may occur and give advantage to the cell, which then undergoes transformation into a LSC and initiates leukaemia. These observations are consistent with the model of pre-tumour progression proposed by Calabrese *et al* (2004) that during the period preceding tumorigenesis, neutral mutations accumulate in stem cells without apparent effects on cell morphology or tumorigenesis. Since the stem cell population is very small, benign mutations can be maintained and accumulated over time due to genetic drift. This “pre-tumorigenesis process” may start from birth and proceed until the tumour formation, a process that can take years or even decades. The shift from the pre-tumour progression towards the tumour progression is marked by a combination of mutations that allows the stem/progenitor cells to undergo transformation and induce tumour formation in a multistep process [69]. Therefore, the early stage of this process is characterized by the formation of a pre-CSC. Shlush *et al* (2014) and Corces-Zimmerman *et al* (2014) have recently identified pre-leukaemic HSCs as chemoresistant HSCs containing mutations arising early in the evolution of AML [109,110]. Such cells are distinct from HSCs but antecedent to leukemic cells and contain some, but not all, leukaemia-specific mutations where the earliest are in “landscaping” genes, genes implicated in the regulation of the epigenetic processes, and the latest are in “proliferative” genes [109,110]. These stem/progenitor cells can acquire extra genetic/epigenetic alterations that can lead to genome instability [15,69].

Telomere attrition or gaining of oncogenic events (e.g. BCR-ABL or AML1-ETO translocations in HSCs) are examples of such events that allow the selection of these cells among others [14,15,68]. Genome instability can lead to additional alterations, such as increased telomerase activity, and inactivation of tumour suppressor genes (e.g. p16^INK4A^, pRb, p53 and/or PTEN), which allow cells to resist to senescence and apoptosis signals and to become immortal [14,68]. These events may lead to the malignant transformation of stem/progenitor cells into CSCs and tumorigenesis. Furthermore, the self-renewal properties of stem cells allow the propagation of these genetic and epigenetic events to their progeny, explaining why non-stem cells (e.g. progenitor cells) can undergo transformation into CSCs [14,68]. Taken together, telomerase activity and/or activation of oncogenes and inactivation of tumour suppressor genes are important factors for the transformation of stem/progenitor cells into CSCs.

Telomerase expression is not homogeneous in all cells within an organism. Indeed, adult stem cells, as ESCs, germ cells and cancer cells, express telomerase, otherwise absent in somatic cells [111,112]. Telomeric DNA extension and maintenance are ruled by telomerase, a ribonucleoprotein that uses an internal RNA subunit as a template, avoiding telomeres shortness and related chromosome instability and aneuploidy associated with loss of cell viability through cell cycle arrest and apoptosis or necrosis [12,113]. Unlike germ cells, telomerase expression in adult stem cells decreases with age, causing telomere shortening with each cell division [114]. When telomeres become too short, a p53-mediated DNA-damage response becomes activated, leading to cell cycle arrest or apoptosis. In fact, downregulation of the tumour suppressor protein p53 in telomerase-deficient mice leads to carcinogenesis [115]. Therefore, telomerase has an important protective activity from genome instability and cancer. On the contrary, these defence mechanisms can lead to organ aging and failure as a consequence of stem cells loss, especially in tissues with high turnover. Tissues with high potential for longevity possess stem cells with longer telomeres and this influences the number of cell divisions a stem cell can undergo [116]. CSCs can therefore be generated from stem cells with short telomeres resulting in genomic instability and accumulation of mutations in tumour suppressor genes or in oncogenes. Consequently, telomerase activity can be increased in CSCs, allowing telomere maintenance and elongation in CSCs and tumorigenesis [111]. Telomerase has being studied in different mouse models of tumorigenesis and studies during aging. These studies showed an onset of age-related diseases related with telomerase-deficiency, for the exception of carcinogenesis. In fact, tumour occurrence was reduced even in the absence of the main tumour-suppressor pathways, except when p53 was downregulated. Higher occurrence of cancer was described when telomerase was overexpressed [12]. Telomerase activity is reactivated in most cancers (85-90%), but when this system is inhibited or is lacked (10-15% of the tumours), tumours express a telomerase-independent system, the alternative lengthening of telomeres (ALT) [117,118]. ALT system is based on the copy of a telomeric DNA template through a homologous recombination and was demonstrated to be expressed in many tumours, being rare in carcinomas [118].

As discussed earlier, inactivation of tumour suppressor genes (e.g. *p53*, *PTEN*) together with activation of oncogenes (e.g. *Ras*, *Bmi1*, and *c-myc*) play an important step in the formation of CSCs and in tumorigenesis [7,11,13,14,68,92,93]. Tumour suppressor genes are well-known DNA damage-induced pathways’ components that promote cell senescence, apoptosis, or transient cell cycle inhibition through the upregulation of specific genes [119]. Downregulation of p53 has been observed in many types of cancer [68]. When its activation is stimulated by stress (e.g. damaged DNA, UV, and oncogenic events), this transcription factor induces the expression of various target genes implicated in several cellular pathways, such as the ones associated with cycle regulation, genome stability, and induction of cell differentiation [120]. Moreover, when activated in stem/progenitor cells, p53 may induce their apoptotic death by stimulating pro-apoptotic proteins (like Bak proteins) or by the down-regulating anti-apoptotic factors (such as Bcl-2 and Bcl-xL) [14]. Instead, when down-regulated due to inactivating mutations, deletion of the p14^ARF^ gene, or through MDM2 gene multiplication in the genome, p53 provides an advantage to cells as they resist to apoptosis, leading to their immortality by unlimited cell divisions [120]. It has been demonstrated that p53 suppression may induce CSC expansion and tumour formation. Therefore, therapies that enhance p53 may lead to the elimination of this type of cells and the inhibition of cancer progression [119].

In addition to p53, PTEN and the corresponding abnormality activated PTEN/PI3K/AKT signalling pathway have been related with cancer. *PTEN*, a tumour suppressor gene that controls cell growth, migration, death and differentiation through AKT regulation [121], can undergo a decrease of its activity through mutations, deletions or methylation silencing of its promoter [122]. Indeed, when depleted in HSCs, NSCs or prostate stem cells, PTEN can lead to leukaemia [123], brain or prostate tumorigenesis, respectively [121]. Mutations in PI3K and AKT were reported in breast CSCs, but also in other cancers, and might be related with tumour proliferation [124].

The activation of oncogenes has been widely demonstrated in many cancers. The oncogenes *c-myc* and *Bmi1* are such examples [125-127]. Recently, c-myc has been used together with other transcription factors for the generation of iPSCs from differentiated cells [128]. This fact demonstrates its importance for stem cell maintenance, although a role in regulating the balance between self-renewal and differentiation of stem cells has also been described [129]. Indeed, cell proliferation can be promoted via c-myc overexpression through its amplification, translocation or activation of downstream genes [126]. In CSCs, *c-myc* oncogene expression is higher than in other cancer cells and it was essential for CSCs proliferation and survival [130]. Another oncoprotein reported to be implicated in the self-renewal regulation of both normal and cancer stem cells is Bmi1 [131,132]. Bmi1 is usually upregulated in cancer [127] and its expression was shown to be important for cancer initiation and progression, and maintenance of the CSC compartment [133,134]. Another example of oncogenes expressing activating mutations in cancer is the Ras family [135]. This family of GTPases is located at the cell membrane and regulates signal transmission from hormones, growth factors and cytokines receptors. These proteins can regulate cell proliferation, differentiation and death through their influence in some signal transduction pathways (e.g. MAPK and PI3K/AKT) [135]. Activating missense mutations in *Ras* are found in many cancers [125]. Interestingly, different expression levels of K-Ras have diverse effects. For instance, when Ras is activated at endogenous levels, cell proliferation is enhanced, but its overexpression induces cell cycle arrest and senescence [125]. Instead, when K-Ras is absent in ES cells, these cells undergo aberrant transformation and acquire an abnormal self-renewal capacity, demonstrating the tumour suppression protein-like behaviour by promoting tumour formation when absent in stem cells [136].

After CSC formation followed by tumorigenesis, the progression of the disease may go through invasion stages such as metastasis. For this step, unlimited self-renewal properties of CSCs through its deregulation are fundamental [14,68] and may be acquired through the activation of Wnt/β-catenin, EGFR, NOTCH, and/or Hedgehog signalling pathways, leading to cell survival, maintenance, and metastasis [14]. Wnt/β-catenin is an important signalling pathway that regulates stem cell potency of embryonic and adult stem cells but also their commitment and differentiation [137]. If Wnt/β-catenin functionality is decreased, the progenitor compartment is compromised due to a decline of the stem cell self-renewal ability [138]. In contrast, abnormal activation of this pathway through β-catenin expression is related with CSC maintenance and tumour development [77,139-141]. Equally, upregulation of the oncoprotein PLAGL-2 in normal and malignant NSC promotes their self-renewal and proliferation through activation of Wnt/β-catenin signalling [142]. This fact is confirmed by the higher copy numbers of PLAGL-2 in human malignant gliomas and colon cancers [142].

## Age-associated epigenetic events and generation of CSCs

Epigenetics is the study of molecular factors and processes that regulate gene function independently of alterations in the DNA sequence [143]. This process is important for the proper function of different cells in a multicellular organism by controlling the activation or silencing of specific genes. Epigenetic changes occur at multiple levels, such as DNA methylation and histone modifications, both affecting chromatin folding, and non-coding microRNAs [144]. The functions of DNA methylation, or the *de novo* addition or maintenance of methyl groups to CpG sites by DNA methyltransferases (DNMTs), include regulation of gene transcription, preservation of parental imprinting, X-chromosome inactivation, and prevention of homologous recombination and chromosomal instability [145,146]. In addition, the posttranslational modifications of histones, such as acetylation and methylation of lysine residues of histones H3 and H4, control chromatin structure and therefore transcriptional activity [147].

Aging is associated with epigenetic changes. This observation is related to an accumulation of epimutations due to the decrement of the epigenetic control with time [16]. In fact, errors may occur during the DNA methylation maintenance, resulting in genes or genomic regions that undergo hypomethylation or hypermethylation during aging [16,148]. The epigenetic deregulation and consecutive genetic expression variation may therefore induce the onset of human disorders, like neurological diseases and cancer [149].

Generally, cancer epigenomes display global DNA hypomethylation associated with hypermethylation at definite promoters [150]. DNA hypomethylation occurs at repetitive sequences, coding regions and at the promoters of many genes, such as the oncogenes *Ras* and *Maspin*. Furthermore, DNA hypomethylation leads to genome instability through chromosomal reorganizations [151-153]. Age-associated hypermethylation has been observed to occur at CpG islands [154] and at promoters of the key developmental genes [155] and polycomb group target genes (PCGT) [156]. Teschendorff *et al* (2010) demonstrated that methylation at the PCGT promoters can lead to the silencing maintenance in stem cells of usually suppressed genes, which may drive to carcinogenesis as stem cell features are preserved [156]. Furthermore, silencing of tumour-suppressor genes, such as *APC*, *p16^INK4A^*, *p14^ARF^*, etc., has been associated with DNA hypermethylation at the promoters [157].

Feinberg *et al* (2005) proposed a model for the origin of CSCs and therefore carcinogenesis: the epigenetic progenitor model of human cancer [153]. According to this model, cancer arises from stem/progenitor cells by three steps: (1) an epigenetic alteration, (2) a mutation-induced oncogene activation or tumour-suppressor silencing, which can be substituted by epigenetic alterations, and (3) genetic and epigenetic instability. The model, first proposed by Feinberg (2005), is further supported by the observation that cancer cells and ESCs share highly similar epigenetic profiles. Importantly, the notion that epigenetic alterations in stem/progenitor cells are the major driving force for carcinogenesis can explain why most types of cancers arise in the elderly. The case of patients with specific cancer-causing mutations (such as in the *APC* gene in colon cancer patients) are especially interesting because probably age-associated epigenetic changes are required for the transformation process [158].

## Age-associated macromolecule accumulation

Deregulation of proteostasis may be involved in cancer and other human diseases, including neurodegenerative diseases, cystic fibrosis, and type 2 diabetes [159,160]. Proteostasis, or homeostasis of the proteome, is an essential mechanism for cell function and viability that coordinates proteome stability by regulating protein synthesis, localization, and folding, and the removal of misfolded, degraded, or aggregated proteins [161]. These processes require many components that are tightly regulated by the cell: i) ribosomes that govern protein translation [162], ii) molecular chaperones that control protein folding, localization, and prevent undesirable protein aggregation [163], and iii) the autophagy and proteasome systems that remove unneeded, misfolded, modified, damaged, and aggregated proteins, which are not rescued by chaperones or other systems [164,165]. In situations of severe proteotoxic stress, senescence or apoptosis can be induced [166].

Many reports have demonstrated that the functionality and efficiency of proteostasis decline with age as shown by the accumulation of damaged proteins in aged tissues [159,160,166-168]. Such damaged proteins may result from protein misfolding, aggregation, or modification, translation errors, ROS, or from genetic alterations [166,169]. As a consequence, damaged proteins can accumulate within cells with age, instigating cell’s malfunction and death due to membrane damage and formation of toxic aggregates [160,166]. For instance, the occurrence of augmented protein modifications, e.g. oxidation, glycation, or carbonylation, with age may disturb several cellular processes, like energy metabolism and protein synthesis, folding and degradation pathways [170]. Therefore, proper maintenance of protein homeostasis in stem cells is of vital importance as it maintains the cell’s proteome and therefore its functionality [159]. Vilchez *et al* (2013) proposed a model to clarify the role of proteostasis in the different types of stem cells and differentiated progeny. According to this model, both long-term stem cells (e.g. HSCs) and differentiated cells (e.g. neurons or cardiomyocytes) possess high-quality proteostasis, decreasing the effects caused by damaged proteins and allowing their long-term survival [160].

Therefore, the temporal accumulation of damaged proteins may occur due to the deregulation of proteostasis [159,160,166,167]. In such conditions, the transcription factor heat shock factor 1 (HSF1) becomes activated and induces the expression of heat shock proteins (Hsps) that undertake the folding of the misfolded proteins and prevent their aggregation [167]. HSF1 has been described to have an important role in carcinogenesis as shown by its role in tumour initiation and progression through the regulation of the expression of Hsps and other targets [171,172]. Indeed, *Hsf1^-/^*^-^ mice expressing a mutant p53 are incapable of forming tumours [171]. Furthermore, HSF1 expression is important for proliferation of cancer cells [173], as demonstrated by a study showing that higher HSF1 expression in breast tumours is associated a poorer prognosis in breast cancer [174]. The importance of HSF1 in carcinogenesis may be associated with its capacity to regulate additional transcriptional programs distinct from heat shock, and therefore protein folding-unrelated, like energy metabolism, DNA repair, apoptosis, etc. The activation of these pathways by HSF1 facilitates malignant transformation, cancer cell survival, and proliferation [172]. HSP90, a target of HSF1, is a molecular chaperone that supports protein folding and prevents their aggregation [175]. This proteostasis factor may support the acquisition of genetic diversity of proteins [159]. Indeed, HSP90 seems to protect and maintain functionality of misfolded proteins that result from destabilizing mutations [159]. Such proteins, like p53, Akt, Bcr-Abl, among others, have important roles within the cellular pathways, and were found to be mutated in cancers [176,177]. HSP90 may have an important role in the survival of CSCs and cancer cells, as demonstrated by the development of cancer therapies targeting this molecular chaperone [178].

If chaperones cannot repair damaged or misfolded proteins, these proteins undergo degradation through autophagy or proteasome [160]. Autophagy, a lysosomal catabolic pathway important for the degradation of damaged organelles and proteins, is an essential physiological mechanism for self-renewal and differentiation of adult stem cells [164,179,180]. Indeed, the long lifespan and the quiescent state of these cells limit their capacity to dilute “cell waste” through their progeny [168]. When this mechanism was absent in stem cells, a block of the differentiation potential together with a loss of pluripotency and self-renewal was observed in these cells [179]. Importantly, autophagy can circumvent oncogenesis by eliminating damaged mitochondria, that would otherwise lead to bioenergetic deficiency, an escalation of ROS levels, and oncogenic proteins like p16/SQTM1 [168]. Autophagy can be suppressed by several oncogenes (e.g. Akt, PI3K, etc.) and be induced by tumour suppressor proteins, such as PTEN, DAPK1, etc.) [168].

The proteasome system and autophagy are important cellular mechanisms that regulate an appropriate protein concentration within the cell [160,165]. Such proteins are involved in many cellular pathways, such as cell cycle, apoptosis, signal transduction, etc. [181]. Although the importance of these systems for hESC pluripotency are known, their role in adult stem cells still needs to be clarified [160].

Taken together, adult stem cells maintain cytoprotective mechanisms to prevent the accumulation of proteins that could otherwise lead to cellular damage. The deregulation of some of the proteostasis pathways, such as autophagy and molecular chaperones, might be related with tumorigenesis. It will be important to understand the role of proteostasis in stem cells during aging and their relation with the generation of CSCs. It is clear that proteostasis is an important process for both CSCs and cancer cells, in terms of CSC maintenance [182-184], chemoresistance [185], migration and invasion [186] and as demonstrated by its activation in cancer cells [171,172,187].

## Conclusions

In the last decade, significant progress has been made in the field of CSC biology and we are gradually gaining a better understanding of the role of these cells in tumour initiation and progression. Indeed, CSCs are cells with stem cell-like properties that may play a central role in the process of carcinogenesis. They have been implicated in tumorigenesis, angiogenesis, invasion and metastasis, and tumour recurrence after therapy. Recent cancer therapies have been focused on CSCs in the hope that their elimination will allow the elimination of self-renewal tumour cells and therefore the bulk tumour, leading to a long-term suppression of disease recurrence.

Although our knowledge in terms of the origin of CSCs has been improved in recent years, there is still much that remains unknown. Different models have been developed supporting the emerging consensus that CSCs arise from a stem/progenitor through temporal accumulation of genetic/epigenetic alterations [14,15,69,95,153]. Indeed, the complex transformation process of stem/progenitor cells into CSCs may involve different steps and it may occur in parallel with a deregulated microenvironment, associated with telomere attrition, inactivation of tumour suppressor genes and upregulation of oncogenes together with genomic and epigenetic instability. At more advanced stages of malignant transformation CSCs may evade the immune system, induce angiogenesis, and finally acquire metastatic capacities.

Due to the fact that age-related effects on stem/progenitor cells are difficult to study experimentally, many aspects of this process are still not well known; especially with regards to the transformation mechanism leading to the generation of CSCs. Therefore, it is of special importance to fill-in the gap between age-associated effects on stem/progenitor cells and tumorigenesis. Likewise, further characterization of the mechanisms involving proteostasis in stem cells and their role during aging is needed, as well as their relation with the development of CSCs.

Therefore, in the context of aging, genetics, epigenetics and the microenvironment are all interconnected, permitting tumour initiation and progression. It is of enormous importance to study the biological properties of CSCs, particularly genotypic and phenotypic features that distinguish them from normal stem cells. Knowledge gained from such studies is a pre-requisite for the development of new therapies that selectively target CSCs, neutralizing their metastatic potential and thus eliminating one of the most lethal age-related diseases, cancer.

## Abbreviations

AML: Acute myelogenous leukaemia; ATRA: All-trans retinoic acid; ALT: Alternative lengthening of telomeres; CSC: Cancer stem cell; CLL: Chronic lymphocytic leukaemia; DNMT: DNA methyltransferase; ESC: Embryonic stem cell; EMT: Epithelial to mesenchymal transition; FACS: Fluorescence-activated cell sorting; HSF1: Heat shock factor 1; Hsp: Heat shock protein; HSC: Hematopoietic stem cell; hPSC: Human pluripotent stem cell; iPC: Induced pluripotent cancer cell; LSC: Leukemic stem cell; miPSC: Mouse induced pluripotent stem cell; NSC: Neural stem cell; NOD/SCID: Non-obese diabetic/combined immunodeficiency disease; OPC: Oligodendrocyte precursor cell; PNS: Peripheral nervous system; PCGT: Polycomb group target; RNS: Reactive nitrogen species; ROS: Reactive oxygen species; SAHA: Suberoylanilide hydroxamic acid

## Competing interests

The authors declare that they have no competing interests.

## Authors’ contributions

SSF wrote the manuscript. HR, JK, MHA, AM, and AD edited the final version.

All authors read and approved the final version.

## Declarations

Publication charges for this article have been funded by the Center of Excellence in Genomic Medicine Research (CEGMR), King Abdulaziz University, Jeddah, 21589, Kingdom of Saudi Arabia.

This article has been published as part of *BMC Cancer* Volume 15 Supplement 1, 2015: Selected articles from the 2nd International Genomic Medical Conference (IGMC 2013): Cancer. The full contents of the supplement are available online at http://www.biomedcentral.com/bmccancer/supplements/15/S1
